# Cellular uptake pathways of sepiolite nanofibers and DNA transfection improvement

**DOI:** 10.1038/s41598-017-05839-3

**Published:** 2017-07-17

**Authors:** Fidel Antonio Castro-Smirnov, Jeanne Ayache, Jean-Rémi Bertrand, Elodie Dardillac, Eric Le Cam, Olivier Piétrement, Pilar Aranda, Eduardo Ruiz-Hitzky, Bernard S. Lopez

**Affiliations:** 1 CNRS UMR 8200, Gustave-Roussy, Université Paris-Saclay, team labeled “Ligue 2014”, 114 rue Edouard Vaillant, 94805 Villejuif, France; 2 Universidad de las Ciencias Informáticas, Carretera a San Antonio de los Baños, km 2 1⁄2, La Habana, 19370 Cuba; 3 CNRS UMR 8126, Gustave Roussy, Université Paris-Saclay, 94805 Villejuif, France; 4 Vectorology and Anticancer therapies, CNRS UMR 8203, Gustave Roussy, Université Paris-Saclay, 94805 Villejuif, France; 5 Instituto de Ciencia de Materiales de Madrid, CSIC, c/ Sor Juana Inés de la Cruz 3, 28049 Madrid, Spain

## Abstract

Sepiolite is a nanofibrous natural silicate that can be used as a nanocarrier because it can be naturally internalized into mammalian cells, due to its nano-size dimension. Therefore, deciphering the mechanisms of sepiolite cell internalization constitutes a question interesting biotechnology, for the use of sepiolite as nanocarrier, as well as environmental and public health concerns. Though it is low, the perfectly stable and natural intrinsic fluorescence of sepiolite nanofibers allows to follow their fate into cells by specifically sensitive technics. By combining fluorescence microscopy (including confocal analysis), time-lapse video microscopy, fluorescence activated cell sorting and transmission electron microscopy, we show that sepiolite can be spontaneously internalized into mammalian cells through both non-endocytic and endocytic pathways, macropinocytosis being one of the main pathways. Interestingly, exposure of the cells to endocytosis inhibitors, such as chloroquine, two-fold increase the efficiency of sepiolite-mediated gene transfer, in addition to the 100-fold increased resulting from sepiolite sonomechanical treatment. As sepiolite is able to bind various biological molecules, this nanoparticulate silicate could be a good candidate as a nanocarrier for simultaneous vectorization of diverse biological molecules.

## Introduction

Sepiolite is a natural and abundant magnesium silicate belonging to the clay mineral family, which constitutes a potential promising nanocarrier for non-viral transfer of biomolecules. Clay minerals is one of the most abundant groups of inorganic solids interacting with the biosphere^1, 2^. Noteworthy, they have been proposed to favors prebiotic reactions of biomolecules, at the origin of life^3^. Because of their physico-chemical characteristics, clay minerals nanoparticles allow the association with biopolymers creating bionanohybrid materials^4–9^, representing thus enticing prospects for biomedical applications^10–18^.

Sepiolite presents a microfibrous morphology^2, 19–22^ showing a promising potential as nanocarrier for non-viral transfer of biomolecules^11, 18, 23^. The theoretical unit cell formula of this silicate is Si_12_O_30_Mg_8_(OH,F)_4_(H_2_O)_4_·8H_2_O)^20, 22^ offering a large surface covered by hydroxyl groups (Si-OH, silanols)^19, 21^ and low cation exchange capacity^2, 19^. These characteristics identify sepiolite as a potential platform for the co-delivery of different kinds of active species. Indeed, sepiolite interacts with polysaccharides^24, 25^, lipids^26^, proteins^27–29^ and virus particles^14, 30^, sleading to a significant diversity of bionanocomposites^10^. Recently, we have proved and characterized the physico-chemical interactions between sepiolite and DNA molecules, using different characterization techniques. We have shown that sepiolite was able to spontaneously transfer DNA into mammalian cells^23^, opening new perspectives for the design of novel strategies for gene therapy and/or the development of new biological models of interest for both academic and applied medical, biotechnological and agronomic research.

Sepiolite-mediated DNA transfer into bacteria requires the friction forces of the so-called Yoshida effect^31, 32^. However, Yoshida effect cannot work with mammalian cells because they would not survive to such treatment. In addition, the microfibrous nature of sepiolite might alarm on any potential asbestos-like effect. These concerns relate to both the use of sepiolite in nanotechnology as well as environmental considerations because sepiolite is present in natural deposits such as for example in Taxus Basin in Spain, and because sepiolite is commonly employed in many uses including domestic, industrial uses, decontamination processes, and pharmaceutical formulations^33, 34^. However, epidemiological studies as well as *in vitro* and *in vivo* analyses, conclude that sepiolite does not represent a health risk^30^, particularly those showing <5 μm fiber length^35^. Consequently, sepiolite is classified as non-hazardous and non-carcinogenic by International Agency of Research on Cancer (IARC)^36^. One hypothesis to account for these results would imply a size effect: the small size of sepiolite would also permit the exclusion of sepiolite from the cells, avoiding a potential asbestos effect. Indeed, using transmission electron microscopy (TEM) analysis, we have previously show that sepiolite fibers from Vallecas-Vicalvaro deposits near Madrid exhibit a mean width of 15 nm, and that 80% of fibers were between 200 and 400 nm long, with a maximal length of 800 nm^23^. Collectively, these data raises the question of the mechanisms of spontaneous uptake into mammalian cells, for the potential hazards on a public health perspective, and with the hope to propose strategies improving DNA transfer efficiency into mammalian cells.

By combining fluorescence microscopy, TEM, time-lapse video-microscopy and fluorescence-activated cell sorting analyses, we show here that sepiolite can be spontaneously internalized and externalized into mammalian cells through both endocytic and non-endocytic pathways. Finally, destabilization of the endosome membranes with chloroquine two-fold stimulated DNA transfer efficiency into human cells.

## Results and Discussion

### Spontaneous internalization of sepiolite into mammalian cells

**Figure 1:**
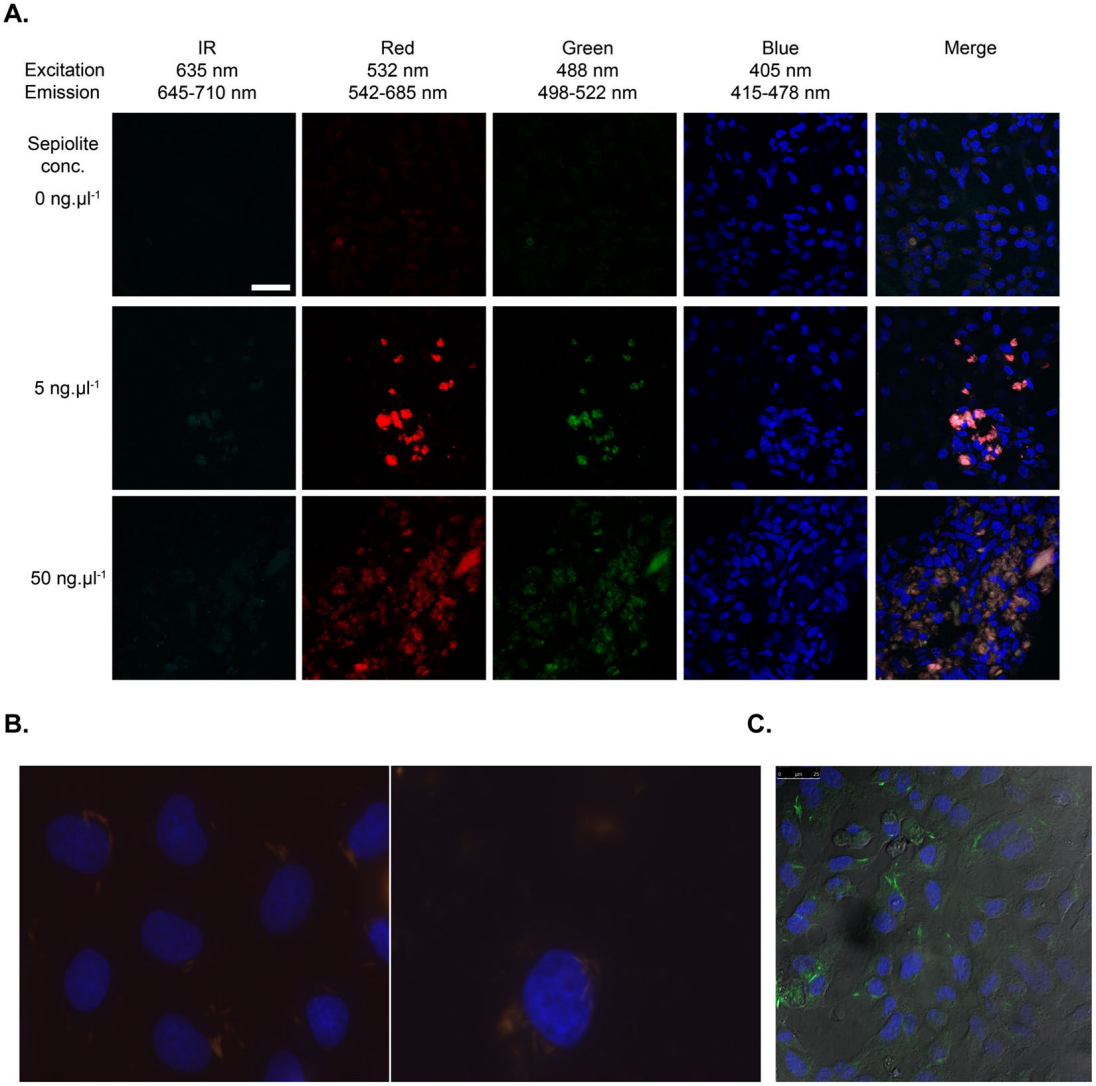
Laser confocal microscopy images of sepiolite fibers (0, 5 and 50 ng·μl^−1^) in V79 cells. (**A**) Channels: IR (infrared), red, green and blue, at given excitation and emission wavelengths. Sepiolite has a natural fluorescence in the green and red ranges and is not fluorescent in blue or infrared. The blue fluorescence represents the Dako-DAPI staining of the cell nuclei. In merged images, we confirmed that sepiolite fibers were uptaken by the cells. IR: infrared. The sepiolite concentration is indicated on the figure. The wavelengths of excitation (Exc) and fluorescence emission (Emis) are indicated. (**B**) Magnified fluorescence microscopy images of sepiolite fibers in V79 cells. Nucleus in blue (DAPI staining), sepiolite fibers in orange/red. (**C**) Phase contrast in confocal analysis visualizing cell contour and the presence of sepiolite (green) inside the cell.

In this work was used a commercial sepiolite obtained from the Vallecas-Vicalvaro deposits near Madrid, Spain (see Experimental Section). Using laser confocal microscopy, we observed a spontaneous fluorescence for sepiolite fibers in mammalian cells: in green, excitation at 488 nm and emission in 498 – 530 nm; in red, excitation at 532 nm and emission in 542 – 685 nm. Therefore, taking advantage of this natural fluorescence, we monitored spontaneous uptake of sepiolite nanofibers into mammalian cells. Although sepiolite fluorescent is low, it is sufficient to detect sepiolite fibers inside the V79 cells by laser confocal fluorescence microscopy analysis (Fig. 1A). In the majority of cases, sepiolite was found in the cytoplasm and close to the nucleus (Fig. 1B,C).

**Figure 2:**
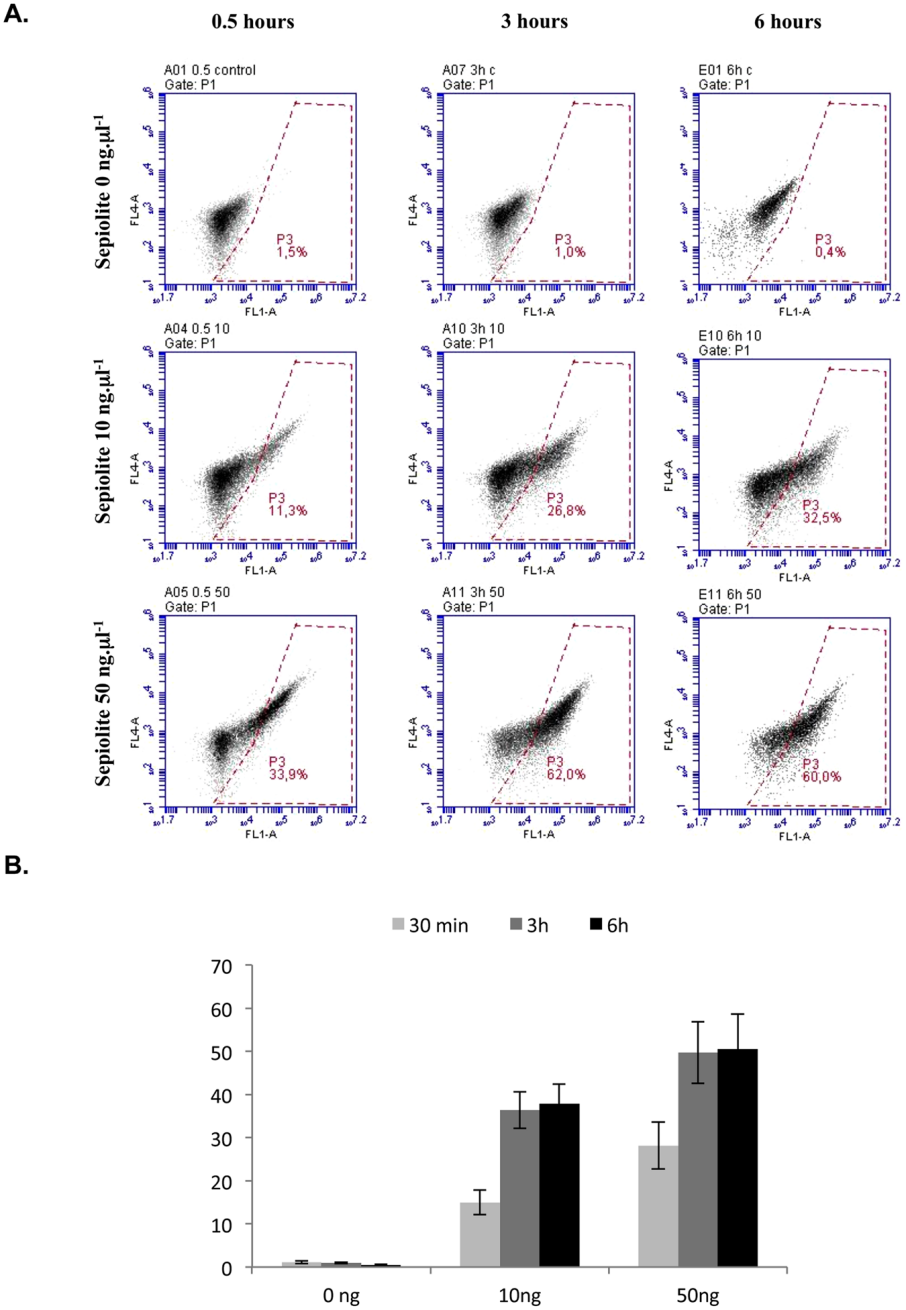
FACS analysis of sepiolite interaction with V79 cells. (**A**) FACS analysis, 0.5, 3 and 6 hours after the addition of sepiolite into V79 cells at different concentrations (0 ng·μL^−1^, 10 ng·μL^−1^, and 50 ng·μL^−1^). The zone P3 corresponds to green fluorescent cells (channel FL1A, 530 nm) from the presence of sepiolite into the cell. 10 000 cells were counted in all experiments. (**B**) Quantification of sepiolite/cell interactions. Values represent the mean +/− SEM of 6 independent experiments.

The kinetics of sepiolite uptake into V79 hamster cells was then determined using fluorescence-activated cell sorting (FACS) analysis, which monitors single fluorescent cells upon contact with the naturally fluorescent sepiolite (Fig. 2). After 30 minutes of contact with sepiolite (10 ng·μL^−1^), 15% of the cells had become fluorescent. These values increased to 36% and 38% after 3 and 6 hours of contact with sepiolite, respectively (Fig. 2B). With 50 ng·μL^−1^, 28% and 50% of cells became fluorescent after 30 min. and 3 or 6 hours, respectively (Fig. 2B). Quantitatively, these values should be underestimated. Indeed, sepiolite can also be spontaneously excluded from the cells (see below). Consistently, using fluorescent-RNA/sepiolite biohybrid, we previously showed that the fluorescent RNA was present in almost 90% of the cells^23^. Therefore the present data should represent the resultant of spontaneous uptake *versus* exclusion.

**Figure 3:**
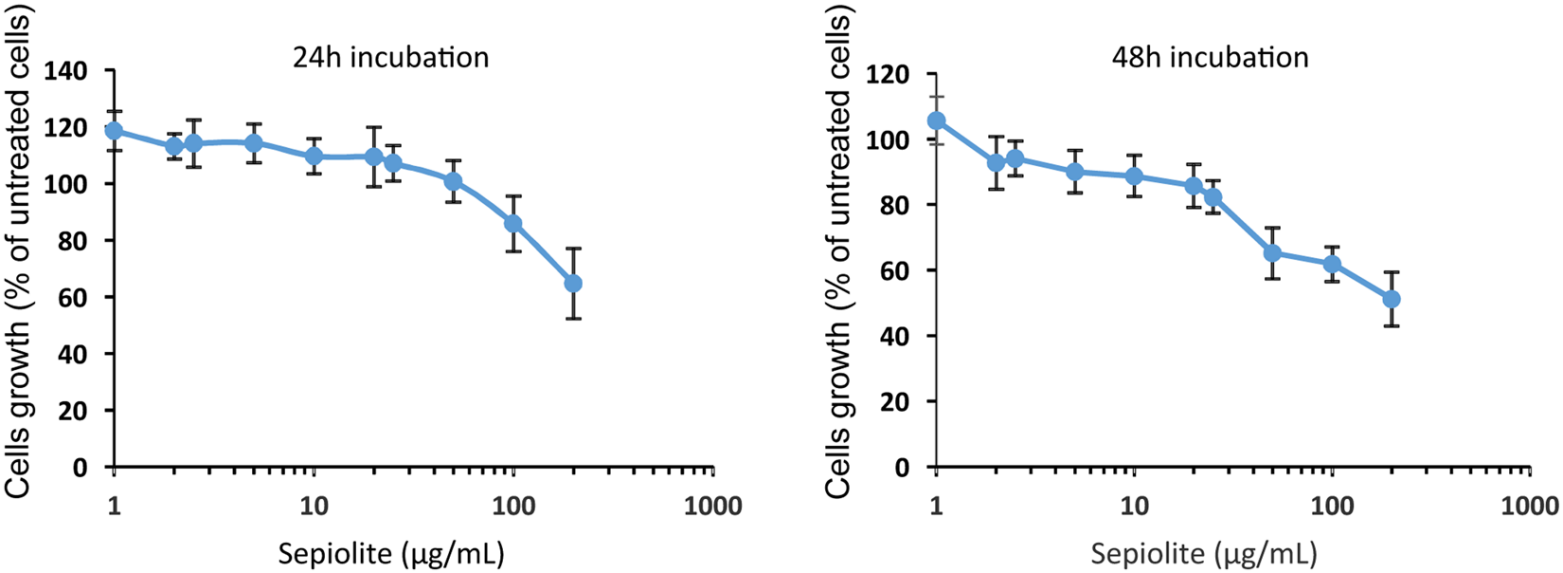
Toxicity of sepiolite. U2OS cells were incubated with increasing quantity of sepiolite for 24 h (left panel) or 48 h (right panel) before MTT test was performed. Each measurement was performed as 8 replicates, and error bare are standard deviation.

The spontaneous uptake of sepiolite fibers into cells was then followed by time-lapse video microscopy (Supplementary data S1). Remarkably, this analysis followed the spontaneous uptake of sepiolite fibers into mammalian cells and revealed that sepiolite fibers can be transmitted from one cell to a neighbor one (Supplementary data S1). Importantly, this analysis also revealed the spontaneous exclusion from the cell (Supplementary data S1), giving support to the absence of substantial toxicity already reported^23, 37^. Nevertheless, we verified the toxicity of sepiolite on cell viability in human U2OS cells (Fig. 3). Up to 50 ng·μL^−1^, sepiolite did not affect cell viability, after 24 h of contact (Fig. 3). 100 ng·μL^−1^ of sepiolite or 48 h of contact slightly increased toxicity (80% of survival cell). Therefore, consistently with the exclusion capacities and the published reports^23, 37^, sepiolite is not toxic, or only moderately at higher concentration. Importantly, in the conditions used for DNA transfection (10 ng·μL^−1^), sepiolite concentration did not exhibit cell toxicity, which occurred for concentration upper than 50 ng·μL^−1^ (Fig. 3).

### Transmission electron microscopy analysis (TEM) of spontaneous sepiolite up-take

**Figure 4:**
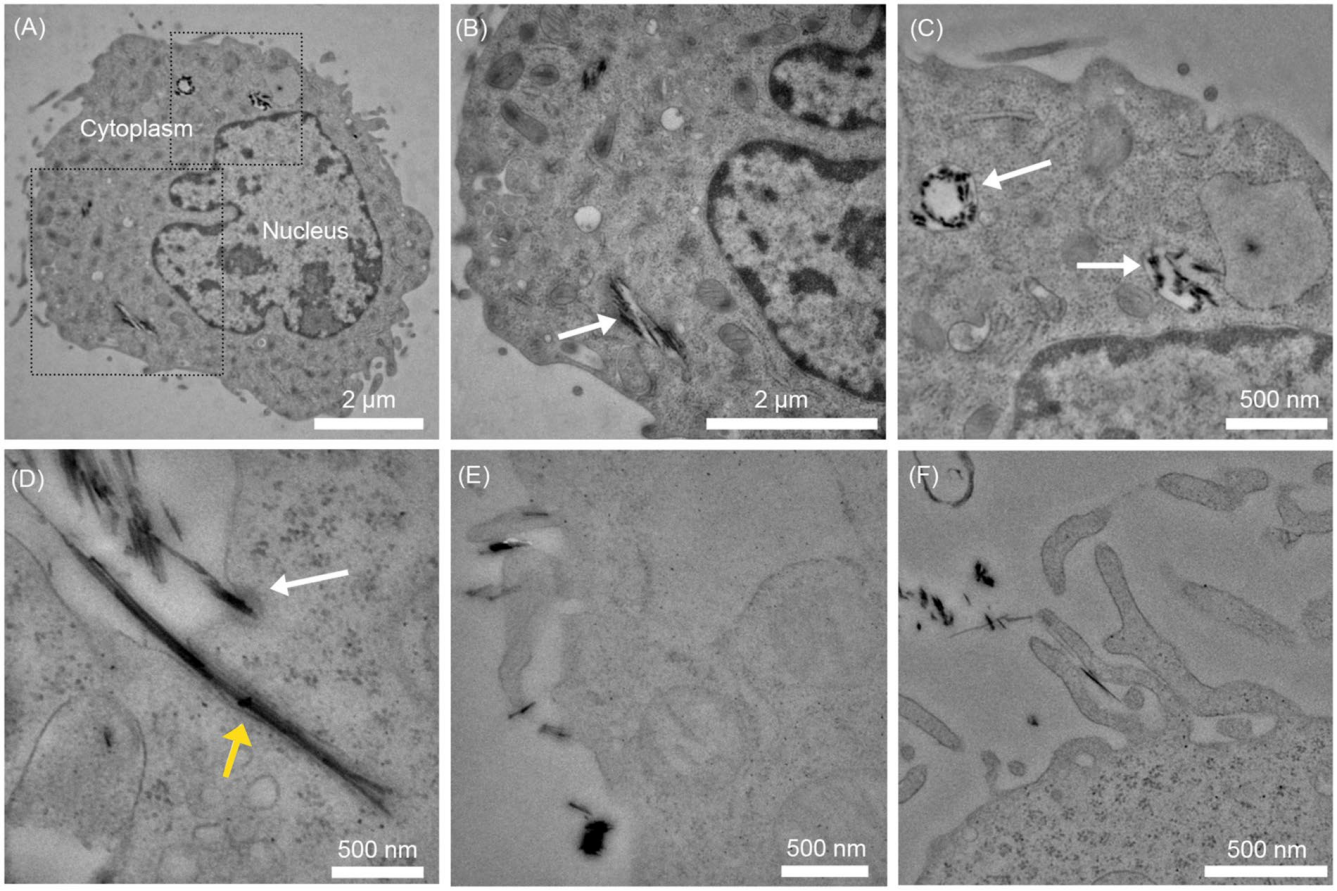
TEM images of the internalization of sepiolite fibers into V79 cells (10 ng·μL^−1^ sepiolite with 5x10^6^ cells). (**A**) A complete image of a cell after 0.5 hour of incubation. (**B** and **C**) A magnified view of a sepiolite fiber in the cell cytoplasm from image A. White arrow shows sepiolite fibers embedded into membranes. (**D**) An image showing endocytosis with membrane invagination (white arrow) and direct fiber insertion (yellow arrow) after 6 h of incubation. **E** and **F**: Images showing the macropinocytosis mechanism for sepiolite uptake in V79 cells.

To detail the cellular structures and mechanisms involved and to increase the resolution of our analysis of the interaction of sepiolite with the V79 cells, we performed a TEM analysis (Fig. 4). We distinguished different steps in the internalization of sepiolite from the membrane surface (see examples Fig. 4D–F) to the inner part of the cytoplasm (see examples Fig. 4A–C). After 0.5 hours of contact, sepiolite fibers were primarily localized at the cell surface; after 1 hour, the sepiolite was localized both at the surface and in the cytoplasm. Finally, after 6 hours of contact, the sepiolite was localized into the cell (Fig. 4A–C).

The TEM analysis also revealed that cytoplasmic sepiolite fibers were embedded within endosome membranes (Fig. 4C), suggesting that fiber entry was the result of endocytosis through membrane invagination (see examples in Fig. 4D white arrow). More specifically, the classical structures of endocytosis and macropinocytosis were observed at the cell membrane/sepiolite junctions (Fig. 4D–F). Macropinocytosis occurs via the formation of actin-driven membrane protrusions, called pseudopods^38^, which are clearly visible here (Fig. 4E and F). These structures fuse with the plasma membrane, leading to internalized sepiolite fibers inside the cytoplasm, surrounded by a membrane (white arrow in Fig. 4C).

It should also be noted that some internalized sepiolite fibers were not embedded within the membranes (see examples in Fig. 4D, yellow arrow), suggesting an additional pathway for sepiolite up-take that is non-endocytic. Direct sepiolite fiber insertion (Fig. 4D, yellow) inside the cells could result from a combination of two factors. The first is related to the particular physical and chemical properties (*i.e*. surface chemistry behavior) of the sepiolite fibers: these properties could allow the fibers to adsorb lipids of the cellular membrane, followed by cellular internalization. Phospholipids, such as phosphatidylcholine, show high affinities to the external surface of sepiolite fibers^15, 26^ giving rise to biohybrid materials that mimic cellular membranes^13, 30^. The second factor is related to the geometry of the sepiolite fibers, which allows for direct insertion, bypassing the classic mechanisms, as observed for other needle-like materials, such as carbon nanotubes^39^. This mechanism was also observed for functionalized carbon nanotubes (CNTs), which exhibited fiber-size and surface charge dependence during the internalization of CNTs into cells^39, 40^.

### Endocytosis inhibitors decreased sepiolite up-take efficiency

To confirm that macropinocytosis and other endocytosis mechanisms are involved in sepiolite internalization into cells, we used FACS to measure the percentage of fluorescent cells following incubation with sepiolite in the presence of endocytosis inhibitors, including chloroquine, which blocks the clathrin mediated endocytosis^41^, and amiloride, which inhibits macropinocytosis^42^. Interestingly, 10 μM of chloroquine reduced the internalization of sepiolite into V79 cells by only 20%, whereas 100 μM amiloride caused a 50% reduction. Importantly, these data show that sepiolite internalization into cells more efficiently results from macropinocytosis, as shown with the TEM observations (see Fig. 4E,F).

### Sepiolite-mediated DNA transfer into human cells, and impact of endocytosis inhibition

**Figure 5:**
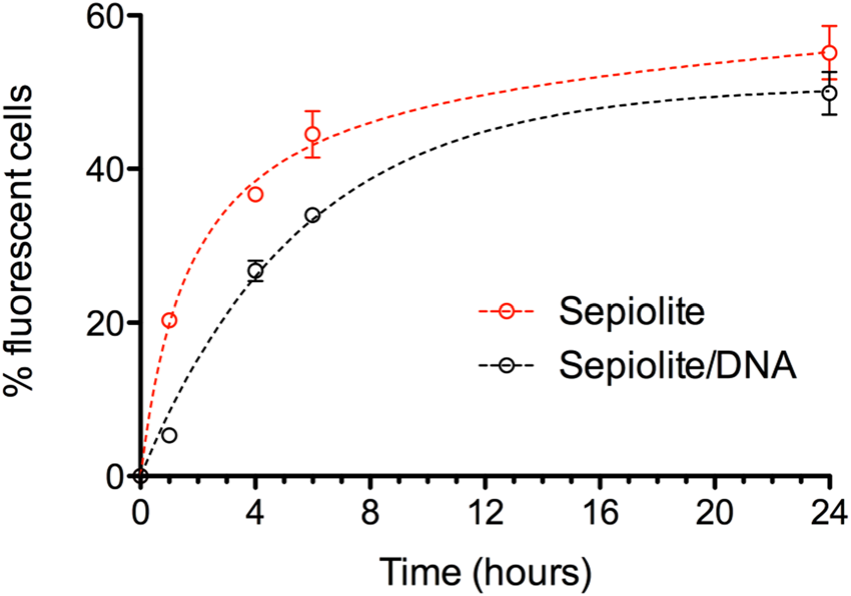
Time kinetics for sepiolite *versus* Sep/DNA interaction with V79 cells. 100 μL of sepiolite dispersion was prepared as following: 45 μL of sepiolite at 2 mg·mL^−1^ and 55 μl of 10 mM TrisHCl pH 7.5. 8.9 ml of cellular medium (MEM) was added and gently homogenized. Finally, 3 mL of sepiolite at 10 ng·μL^−1^ in cellular medium was added in each set of 3 wells with 2x10^4^ of V-79 cells in each well. Cells were collected after trypsinization and analysed by FACS 1 h, 4 h, 6 h, and 24 h after addition of sepiolite. 10,000 cells were counted for each sample.

First we compared the kinetics and efficiency of interaction of sepiolite *versus* a sepiolite/DNA biohybrids (Sep/DNA) by FACS analysis (Fig. 5). The Sep/DNA interaction with cells was delayed compared to that with sepiolite alone, but finally raised the same efficiency (Fig. 5). Indeed, a time-kinetic analysis showed that only 5% of the cells fluoresced after 1 hour of contact with Sep/DNA. However, the uptake of Sep/DNA reached the uptake of sepiolite alone after 6 hours (Fig. 5). These differences in the kinetics might result from the electrical charge alteration revealed by the zeta-potential values due to the binding of DNA onto sepiolite^23^.

**Figure 6:**
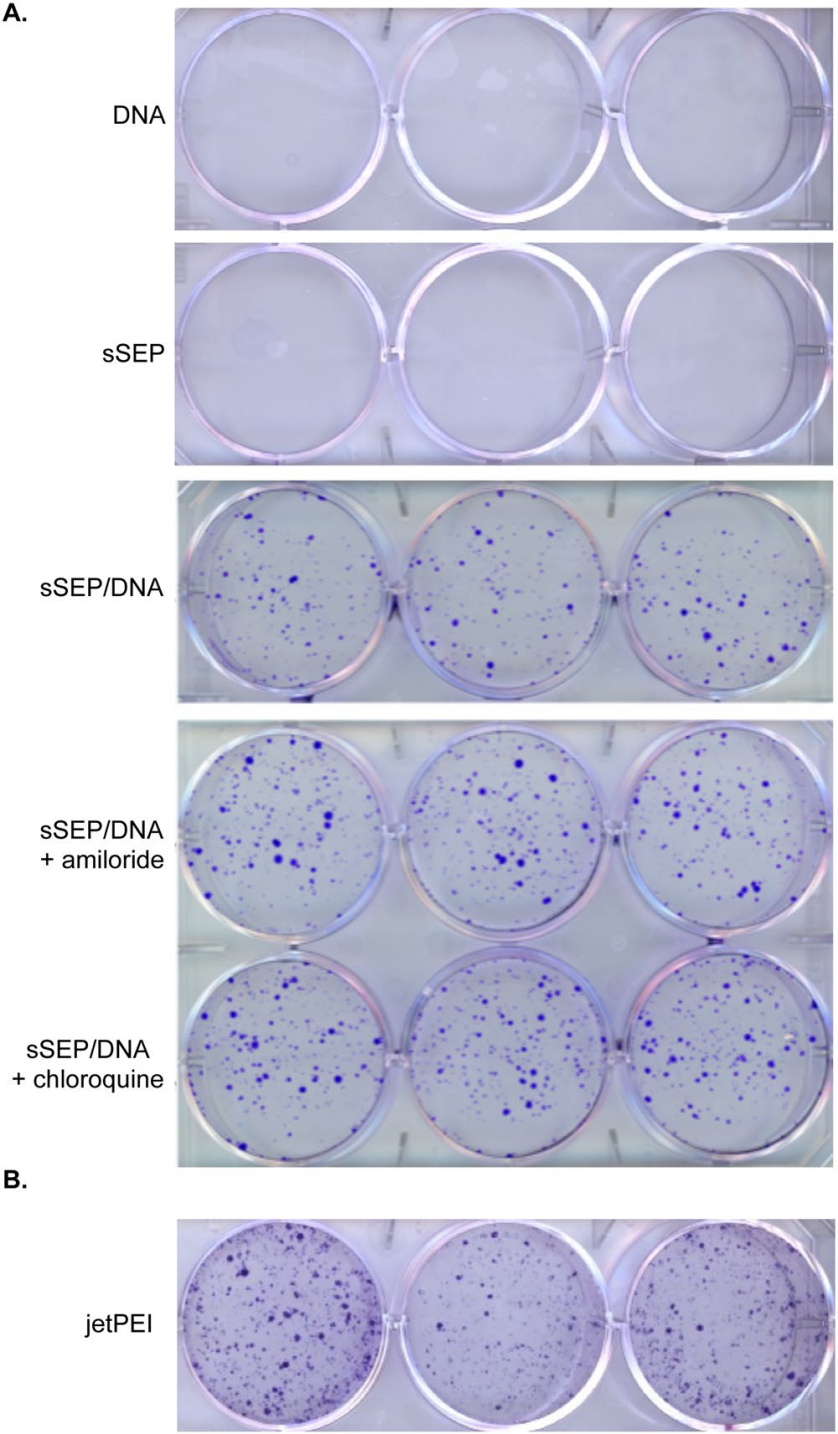
DNA transfer into human cells. **(A**) A comparison of the number of colonies of transfected U2OS human cells with sSep/DNA (triplicates), and sSep/DNA biohybrids after incubation with 100 μM Amiloride (triplicates) and 10 μM chloroquine (triplicates). (**B**) Transfection of U2OS cells using jetPEI.

The DNA transfer efficiency of the Sep/DNA biohybrid into U2OS human cells was further determined using a plasmid DNA encoding for a resistance gene to G418. Transfected cells were selected based on the acquired resistance to G418 as a result of the transferred plasmid bound to the sepiolite. Here we used sonicated sepiolite (sSEP), because we showed that sonication of sepiolite before binding of the DNA strongly increased transfection efficiency^23^. As already shown^23^, stable resistant colonies were obtained, after 10 days of selection with G418, (Fig. 6A).

Transfection involves multiple steps, including cellular binding, internalization and delivery into the cells. Chloroquine, a weak base that can rapidly penetrate the plasma membrane, accumulates in acidic vesicles and increases the pH of the acidic compartiment^43^. The prevention of endosome acidification subsequently inhibits hydrolytic enzymes, such as proteases and nucleases^44^. Additionally, chloroquine causes the swelling and rupture of endosomal vesicles by increasing the osmotic pressure inside the acidic compartment^45^. Because chloroquine can neutralize the acidic compartment and induce the rupture of endocytic vesicles, additions of chloroquine have been shown to improve nucleic acid transfer by other methods^44, 46^. Unfortunately, chloroquine also inhibits the internalization of endogenous molecules by blocking the clathrin mediated endocytosis pathway. Thus, upon chloroquine exposure, the transfection efficiency should result from the balance of two opposite processes, the potential inhibition of cellular uptake *versus* the endosome disruption and protection against nuclease degradation. However, in the present case, chloroquine moderately affected the efficiency of sepiolite uptake (see above). Therefore, the transfection efficiency after the prior incubation of cells with amiloride and chloroquine was investigated. Amiloride only led to a slight increase in the transfection efficiency in U2OS cells (Fig. 6A). Interestingly, treatment with chloroquine led to a two-fold increase in the transfection efficiency into human U2OS cells (Fig. 6A), in addition to the 100 fold increase when using sonicated Sep/DNA^23^. Remarkably, the transfection efficiency with sSEP and addition of chloroquine was close to that using the classical commercial jetPEI (a polyethylenimine derivative) method (Fig. 6B). Thus, under the present conditions, it is clear that chloroquine promotes endosomal escape more efficiently than it inhibits internalization, while the impact on internalization is moderate, consistently with the FACS analysis (see above).

### Concluding remarks and perspective

**Figure 7:**
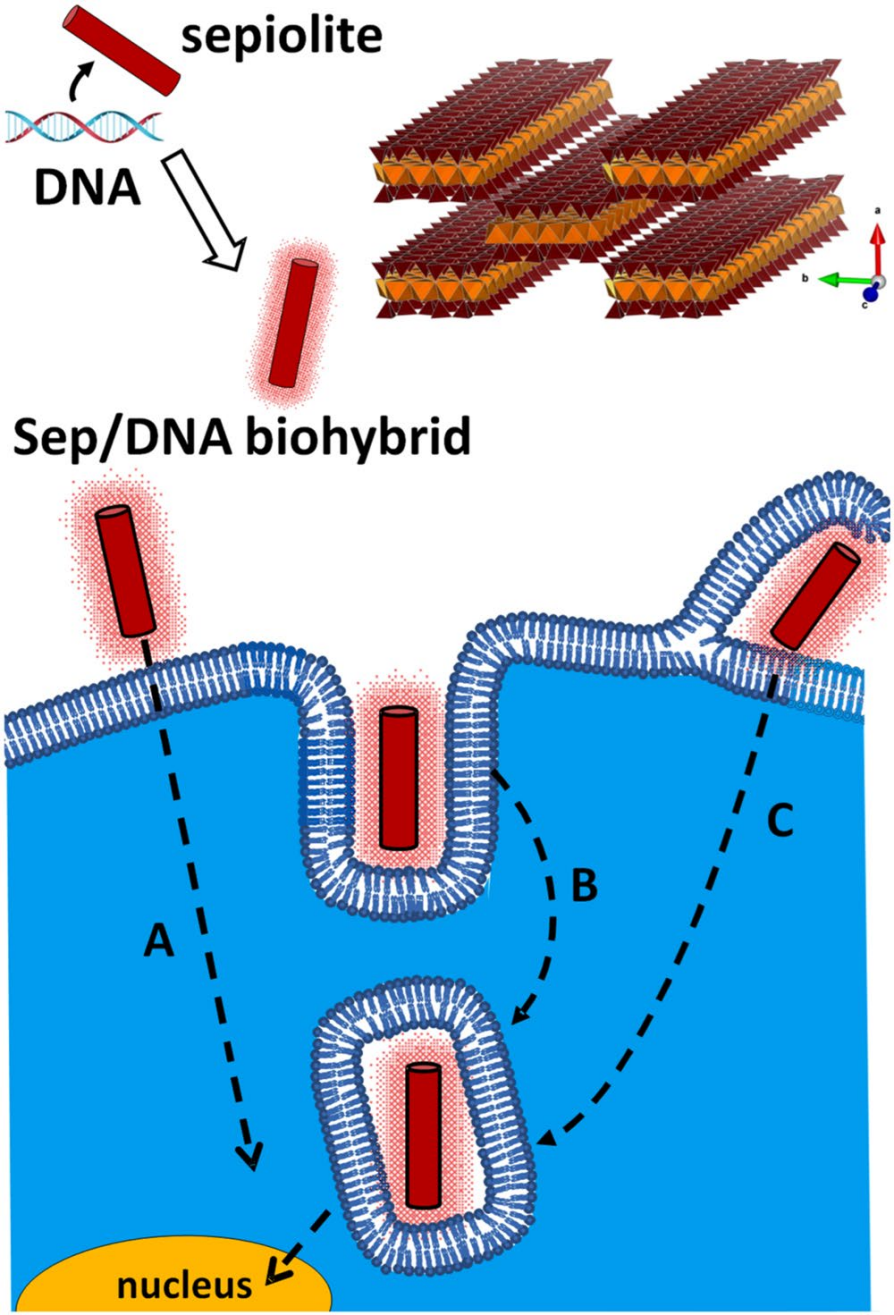
Scheme of sepiolite-mediated DNA delivery into mammalian cells. DNA interacts with sepiolite then the Sep/DNA biohybrids interacts with mammalian cells. The Sep/DNA complex can be internalized into cells either directly (**A**), or via endocytosis through invagination of the cell membrane (**B**), or via macropinocytosis with pseudopods (**C**). These last processes result in embedded Sep/DNA complex into the cytoplasm. In some occasion direct internalization of Sep/DNA result in unembedded complex. After spontaneous release, the DNA is imported to the nucleus leading to its stable integration into the cell genome.

Together, these results demonstrate that sepiolite nanofibers are spontaneously and efficiently internalized by mammalian cells by several processes, but mainly by clathrin-mediated endocytosis and macropinocytosis into cells, favoring the delivery of bound molecules such as DNA (summarized in Fig. 7). Therefore playing on endosomes membranes stability, it is possible to improve sepiolite-mediated DNA transfer efficiency. Altough the efficiency of transfection is slightly lower than using jetPEI, the efficiency is high and we show here that we can increase it. Moreover, sepiolite shows other advantages, such as its low cost and its capacity to bind various biological molecules. Therefore, this nanoparticle constitutes an appealing candidate as a nanocarrier, affording a new approach of vectorization of multiple biological molecules, in contrast with many other methods. Moreover, since we show here that it is possible to detect sepiolite fluorescence by FACS, a promising perspective is the possibility to select cells containing Sep/DNA using cell-sorting techniques.

Finally and importantly, we show here that sepiolite can also be exported from the cell, and, consistently, exhibits a poor toxicity. This capacity should prevent a putative asbestos effect.

## Material and Methods

### Sepiolite

Sepiolite from the Vallecas-Vicálvaro clay deposits (Madrid, Spain) was furnished by TOLSA S.A., being a product of rheological grade commercialized as Pangel S9 (>95% of pure sepiolite). A sepiolite suspension of 2 mg·ml^−1^ was prepared in 10 mM Tris-HCl buffer, pH = 7.5, using a sonicated sepiolite (sSep). Sepiolite suspension was sonicated, 3 times at 30% amplitude for 10 s each time using a Vibra-Cell 75042 from Bioblock Scientific. SSep was then sterilized by autoclaving.

### Cell culture

Chinese hamster cells were grown in dishes as monolayers in modified Eagle’s medium (MEM) containing 10% fetal bovine serum (FBS). U2OS human osteosarcoma cells were grown in dishes as monolayers in Dulbecco’s modified Eagle’s medium (DMEM) from Life Technologies containing 10% (v/v) FBS. Cells were incubated at 37 °C with 5% CO_2_ in air and 95% humidification. MEM, DMEM and FBS were purchased from Life Technologies^TM^.

### Fluorescence microscopy

Cells were grown on coverslips. Cells were washed in PBS and fixed in 4% formaldehyde. Coverslips were mounted in mounting medium (Dako) supplemented with DAPI (Sigma). Images were acquired on an Axio Imager Z1 microscope using the Axio Vision software (Zeiss).

For laser confocal microscopy, images were acquired on a confocal Leica SpE with the 63x objective (ACS AP063.0 × 1.30 oil). The pictures were 1,024 by 1,024 pixels with a pixel size of 174.6 μm.

### FACS experiments

#### Time kinetic for sepiolite uptake in mammalian cells

100 μL of sepiolite dispersion was prepared as following: 45 μl of sepiolite at 2 mg·mL^−1^ and 55 μl of 10 mM TrisHCl. 8.9 ml of cellular medium (MEM) was added and gently homogenized. Finally, 3 mL of sepiolite at 10 nμl^−1^ in cellular medium was added in each set of 3 wells with 2x10^4^ of V-79 cells in each well. Cells were collected after trypsinization and analysed by FACS 1 h, 4 h, 6 h, and 24 h after addition of sepiolite. Samples were analysed with a C6 flow cytometer using the C6 Flow software (BD Accuri), with excitation at 488 nm and emission at 530 +/− 15 nm. 10,000 cells were counted for each sample.

#### Time kinetic for sepiolite/DNA uptake in mammalian cells

100 μL of Sep/DNA dispersion was prepared as following: 45 μL of sepiolite at 2 mg·ml^−1^, 20 μL of 50 mM CaCl_2_, 5 μl of 10 mM TrisHCl pH 7.5 and 20 μL of PUC plasmid at 400 ng·μL^−1^. Then 8.9 ml of cellular medium (MEM) was added and gently homogenized. Finally, 3 mL of sepiolite suspension at 10 ng·μL^−1^ in cellular medium was added in each well with 2x10^4^ of V-79 cells in each well. Cells were collected after treatment with trypsin and analysed by FACS 1 h, 4 h, 6 h, and 24 h after addition of Sep/DNA complex. Samples were analysed by FACS on a C6 flow cytometer using the C6 Flow software (BD Accuri). 10,000 cells were counted for each sample.

#### Endocytosis inhibition

V79 hamster cells and U20S human cancer cells were incubated with 1 mL of 100 μM amiloride at 37 °C^41, 42^. The treatment was performed 30 min before the addition of 10 ng·μL^−1^ sepiolite suspension to the cells. Amiloride HCl dihydrate (Catalog No. S2560) was provided by Selleck Chemicals (www.selleckchem.com), in a 10 mM/1 mL stock solution. The reagent was used for the selective inhibition of macropinocytosis in mammalian cells. For clathrin mediated endocytosis inhibition, V79 hamster cells and U20S human cancer cells were incubated with 3 mL of 10 μM chloroquine at 37 °C. The treatment was performed 30 min before the addition of 10 ng·μL^−1^ sepiolite suspension to the cells. Chloroquine diphosphate salt (C6628-25G lot # BCBK7067V) was supplied by Sigma Aldrich. A 100 mM stock solution was prepared by dissolving 200 mg chloroquine in 3.9 mL water with gentle vortexing and then was filtered through a 0.22 μm filter for sterilization. Measurements were performed by FACS in CyFlow Space from Partec; 10,000 cells were counted for each sample.

### Cell toxicity

To determine the cytotoxicity of sepiolite, 5x10^3^ U2OS cells were seeded in 96-well plate 1 day before treatment. Then, the medium was discarded and 100 mL of fresh medium containing increasing quantity of sepiolite was added. The cells were incubated for 24 h or 48 h, and cell surviving was determined by the MTT test. Then, 10 mL of 5 mg·mL^−1^ MTT (Sigma-Aldrich Chemical Co.) in phosphate-buffered saline (PBS) buffer was added to the cells and incubated for 2 h at 37 °C, 5% CO_2_ in moist atmosphere. Cells lyses and formazan solubilization were obtained by adding 100 mL of 10 mM HCl, 10% sodium dodecyl sulfate (SDS) solution followed by overnight incubation at 37 °C. Produced formazan was quantified by measurement of absorbance at 570 nm and 630 nm in plate reader (EL808; BioTek). Experiments were performed on eight independent wells and expressed as the percent of untreated cells and standard deviation is calculated.

### Cellular TEM imaging

V79 cells were prepared using 2.5% glutaraldehyde fixative solution in 0.1 M cacodylate buffer solution (pH 7.4) for 1 h and then rinsed in the same buffer solution (3 × 5 min). A post fixative step was performed with 1% OsO_4_ in cacodylate buffer, and then, the cells were water rinsed (3 × 10 min). Cells were dehydrated in an ethanol solution (90% at 2 × 10 min and 100% at 3 × 10 min). The substitution was made in epon/acetone solutions in a volume ratio of 1/3, 1/2 and 3/1, with each for 1 h. At the final substitution step, a new bath of epon was administered for 1 h before the last overnight step. Cells were then encapsulated with new epon using a BDMA hardener for polymerization at 60 °C for 24 h. The sample preparation of cell thin sections for TEM observation was performed using a Leica Ultracut UCT ultramicrotome. The experimental conditions were a 35° diamond knife, section thickness of 90 nm and a 1.6 mm/s speed rate. Cell thin sections were deposited on carbon/collodion grids before staining with 2% uranyl acetate solution in water for 20 min and lead citrate for 3 min. To investigate the sepiolite uptake mechanisms in cells, TEM analysis was performed on four types of samples. The sample types corresponded to the cell culture sepiolite incubation times, allowing the detection of the first steps from internalization by the membrane surface to the inner part of the cytoplasm and nucleus, i.e., 0, 0.5, 1 and 6 h. The sepiolite fiber-cell interactions were analyzed by TEM using a 80 KeV 902 Zeiss transmission electron microscope equipped with an electron energy filter in column. Bright-field TEM imaging mode was employed using zero loss energy filtering.

### Cell transfection

Cell transfection was carried out in 6-well plates, with 10^4^ of U2OS human osteosarcoma cells cultured per well, as previously described^23^. Chloroquine (10 μM) or amiloride (100 μM) were added 30 min. before the transfection mix (sSep/DNA). For jetPEI transfection, 10^4^ of U2OS cells per well were transfected with 1 μg of plasmid DNA under the conditions specified by the purchaser (Polyplus, Ilkirch, France).

## Electronic supplementary material

Supplementary Information

Supplementary Information

Supplementary Information

Supplementary Information

Supplementary Information
