# Supplementary material for: Cellular uptake pathways of sepiolite nanofibers and DNA transfection improvement
Source: Sci Rep. 2017 Jul 17;7:5586. doi: 10.1038/s41598-017-05839-3 (PMC5514060; doi:10.1038/s41598-017-05839-3)
Supplement: Supplementary file 1 — Supplementary Information [file 41598_2017_5839_MOESM1_ESM.pdf]

Supplementary data

Cellular uptake pathways of sepiolite nanofibers and DNA transfection improvement

Fidel Antonio Castro-Smirnov, Jeanne Ayache, Jean-Rémi Bertrand, Elodie Dardillac, Eric Le Cam, Olivier Piétrement, Pilar Aranda, Eduardo Ruiz-Hitzky and Bernard S. Lopez.

Supplementary data S1

4 files time-lapse video microscopy

internalization A : Internalization of sepiolite in a V79 cell.

Internalization B : Internalization by endocytosis of sepiolite in V79 cell. The endosome can be seen.

Internalization C: Cell division in presence of sepiolite, sepiolite ejected from the cell and transferred to another cell.

Transfert D : Transfert of sepiolite from one cell to another.

Materials and methods

One day before the acquisition, 10^5 of V79 cells were plated in Lab-Tek Chambered #1.0 Borosilicate Cover Glass System 155380 (2 chambers), in modified Eagle's medium (MEM) containing 10% fetal bovine serum (FBS). Just before the acquisition, the cellular medium was changed with a new MEM with $10ng.\mu l^{-1}$ sonicated sepiolite (sSep). Images were acquired every 6 min 30 sec within 18 hours 20 min on an Olympus Fluoview FV10i with the objective UPLSApo60x.
